# Blood–Brain Barrier Dysfunction in Mild Traumatic Brain Injury: Evidence From Preclinical Murine Models

**DOI:** 10.3389/fphys.2020.01030

**Published:** 2020-08-21

**Authors:** Yingxi Wu, Haijian Wu, Xinying Guo, Brock Pluimer, Zhen Zhao

**Affiliations:** ^1^Center for Neurodegeneration and Regeneration, Zilkha Neurogenetic Institute and Department of Physiology and Neuroscience, Keck School of Medicine, University of Southern California, Los Angeles, CA, United States; ^2^Department of Neurosurgery, Second Affiliated Hospital, School of Medicine, Zhejiang University, Hangzhou, China; ^3^Neuroscience Graduate Program, Keck School of Medicine, University of Southern California, Los Angeles, CA, United States

**Keywords:** mild traumatic brain injury, blood-brain barrier, murine model, vascular link, neurodegenerative diseases

## Abstract

Mild traumatic brain injury (mTBI) represents more than 80% of total TBI cases and is a robust environmental risk factor for neurodegenerative diseases including Alzheimer’s disease (AD). Besides direct neuronal injury and neuroinflammation, blood–brain barrier (BBB) dysfunction is also a hallmark event of the pathological cascades after mTBI. However, the vascular link between BBB impairment caused by mTBI and subsequent neurodegeneration remains undefined. In this review, we focus on the preclinical evidence from murine models of BBB dysfunction in mTBI and provide potential mechanistic links between BBB disruption and the development of neurodegenerative diseases.

## Introduction

Traumatic brain injury (TBI) is a leading cause of death and long-term disability around the world (Hackenberg and Unterberg, 2016). Based on the severity, TBI can be classified as mild, moderate, and severe TBI (Chamelian and Feinstein, 2004). As more than 80% of cases are estimated to be mild cases (Rutland-Brown et al., 2006), it is particularly important to understand the pathophysiological mechanisms of mild TBI (mTBI) and develop novel and effective therapeutic approaches. Accumulating evidence has demonstrated that mTBI can result in a series of pathologic events, including neuroinflammation, oxidative stress (Katz et al., 2015), cerebrovascular impairment such as edema, circulatory insufficiency, and blood–brain barrier (BBB) breakdown (Doherty et al., 2016). These events are highly interactive, and all contribute to the long-term cognitive and emotional impairments in mTBI patients (Riggio, 2011).

The BBB is a highly selective membrane that mainly encompasses endothelial cells, sealed by tight junctions, and fortified by pericytes and astrocytic endfeet (Daneman and Prat, 2015). This coordinated network of cells plays an important role in the brain’s physiological homeostasis and functions, while disruption of this network can trigger multiple pathologic events (Zhao et al., 2015). In fact, BBB dysfunction has been increasingly noticed in many neurological conditions of the central nervous system (CNS), including acute injuries such as TBI and stroke, and chronic neurodegenerative disorders such as Alzheimer’s disease (AD), Parkinson’s disease (PD), and chronic traumatic encephalopathy (CTE) (Sweeney et al., 2019). It is worth noting that BBB dysfunction was commonly observed in both mTBI patients and experimental animal models (Sandsmark et al., 2019). For instance, histological evidence from human patients indicated that microvascular dysfunction widely occurred from mild to moderate and severe TBI, and not only in the acute and subacute stages after the primary injury but also in the chronic stage in long-term survivors (O’Keeffe et al., 2020). These clinical findings are in general backed up by the evidence from preclinical animal models (Sandsmark et al., 2019), which demonstrated that mTBI induces cellular and molecular events at the BBB, including alteration of endothelial transport functions (Villalba et al., 2017), disruption of the crosstalk between endothelial cells and pericytes (Bhowmick et al., 2019), pericyte loss (Zehendner et al., 2015), cerebral blood flow (CBF) reduction, and tissue hypoxia (Han et al., 2020; O’Keeffe et al., 2020). These vascular pathological events interact and evolve with neuroinflammation (Blennow et al., 2012) and contribute to chronic neurodegeneration post-injury.

**FIGURE 1:**
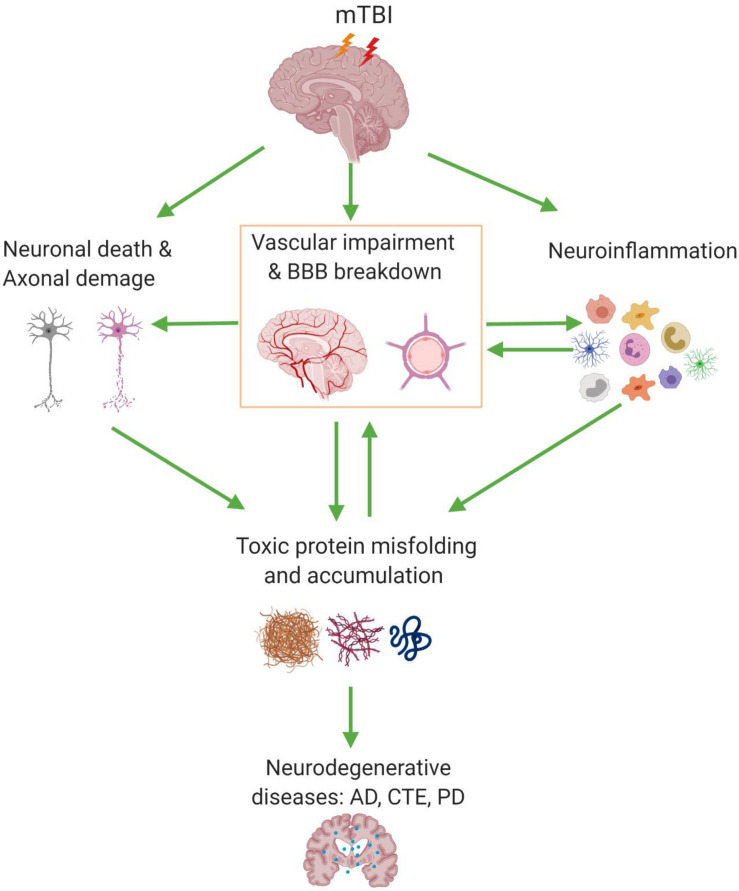
Blood–brain barrier (BBB) dysfunction and its associated pathologic events involved in mild traumatic brain injury (mTBI). mTBI can cause a series of pathologic changes including cerebrovascular impairment and BBB dysfunction, neuronal cell death, and axonal damage, as well as gliosis and neuroinflammation. Also, BBB dysfunction can accelerate the accumulation of pathologic products such as Aβ, tau, and α-synuclein; on the other hand, their deposition around the cerebral vessel has a chronic toxic effect which enhances the BBB disruption and ultimately may lead to neurodegenerative diseases. AD, Alzheimer’s disease; CTE, chronic traumatic encephalopathy; PD, Parkinson’s disease.

More importantly, clinical data indicated that BBB impairment can persist for many years and is highly associated with long-term neurological deficits in mTBI patients (Shlosberg et al., 2010). Therefore, it is crucial to evaluate the extent of BBB disruption after mTBI and elucidate the underlying molecular cascades in preclinical models. Such knowledge will not only define a clear vascular link between mTBI and long-term neurological impairments, as well as build up a foundation for developing novel therapeutic approaches. Animal models, more specifically murine models, often closely mimic key neuropathological features in human patients and allow us to study the underlying mechanisms of BBB dysfunction and its associated pathophysiologies in CNS diseases. Therefore, in this review, we summarize recent evidence in the last 10 years obtained from experimental murine models of mTBI, address the BBB disruptions and its associated pathologic changes in mTBI, and depict the vascular link between mTBI and subsequent neurodegeneration (Figure 1). The criteria used for mTBI (include repetitive mTBI) are mainly based on the recent systematic review (Bodnar et al., 2019). Only studies with histological and/or behavioral validation of mTBI were included to ensure a closer recapitulation of clinical observations under the mTBI category (Bodnar et al., 2019).

## Blood–Brain Barrier Dysfunction in Murine Models of Mild Traumatic Brain Injury

**FIGURE 2:**
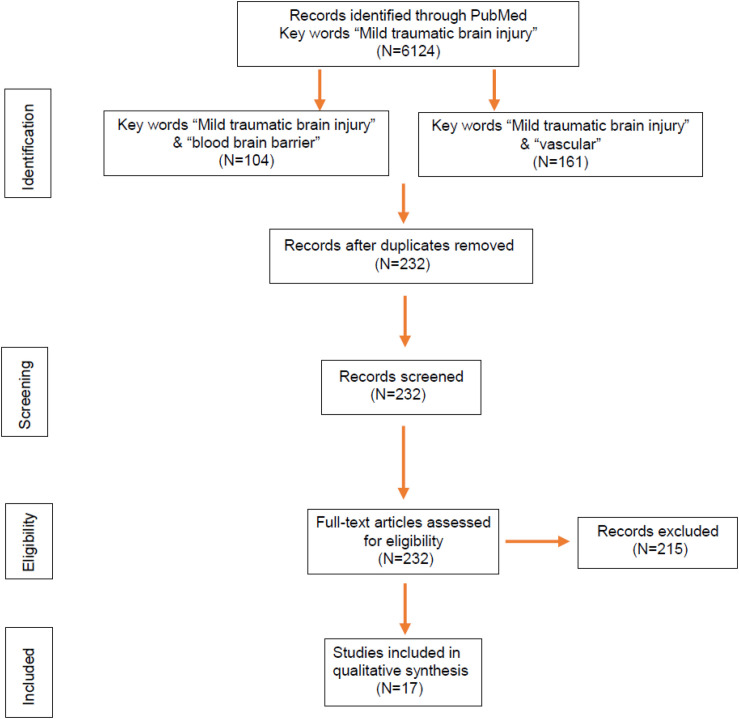
Flowchart of article selection. Identification through PubMed searches yielded 232 articles related to blood–brain barrier (BBB) and vascular impairment in mild traumatic brain injury (mTBI) published during 2010–2020, which were further screened. Based on abstract and eligibility, 215 articles were excluded, and a total of 17 original research articles with BBB dysfunction in murine models of mTBI were determined by full-text examination and included in the review.

Murine models have helped us tremendously to understand the pathogenic events after mTBI, including cerebral microvascular injury and BBB dysfunction. We searched over 6,000 publications related to mTBI on PubMed and found 232 studies potentially covering cerebrovascular impairment and BBB dysfunction (Figure 2). Among them, 17 research articles in the last 10 years were based on murine models of mTBI (Table 1) and selected. The models include not only the well-established weight drop and piston-driven models (Bodnar et al., 2019) but also the increasingly appreciated models including modified controlled cortical impact (CCI), mild blast injury, and fluid percussion (Lifshitz et al., 2016; Bharadwaj et al., 2018). The results were organized by the timing of assessments and mechanism of pathogenesis and discussed in the context of methods used to generate the impact and detection of the vascular impairment.

**TABLE 1:** An update on mTBI studies in murine models in recent 10 years.

Study	Animal	Model	BBB Impairment	Neuropathology	Neuroinflammation	Motor function	Cognitive function
Yang et al., 2013	Mouse	Weight drop	BBB disruption detected by Lucifer yellow and FITC-albumin at 1.5–6 h	Increased expression of serum NSE at 1.5–6 h, 1 day	Increased expression of brain MDC and MIP-1α on 1 day	Deficit in rotarod test on 1–5 days	Impairment (NOR: 1–4 days)
Chen et al., 2014	Mouse	Modified CCI	BBB disruption detected by Evan blue at 1 h	Axonal injury on 1 day; Increased neuronal degeneration on 1–8 days	Activation of astrocytes and microglia on 8 days	Deficit in rotarod test on 1–3 days	No impairment (SOR and contextual fear conditioning: 5–8 days)
Gama Sosa et al., 2014	Rat	Blast exposure; RHI	Altered microvascular ECM, microvascular occlusion and degeneration, intraventricular hemorrhage at 6–10 months	n/a	Activation of astrocytes in 10 months	n/a	n/a
Katz et al., 2015	Rat	LFP	BBB disruption detected by Evan blue at 24 h	n/a	Activation of astrocytes at 24 h; Cytokine upregulation at 24 h	n/a	Impairment in NBS and NSS tests at 2–24 h
Li et al., 2016	Mouse	Micro TBI compression	BBB disruption detected by rhodamine B at 5–60 min	Increased cell death at 6–24 h	n/a	n/a	n/a
Logsdon et al., 2018	Mouse	Blast exposure; RHI	BBB disruption detected by ^14^C-sucrose and ^99m^Tc-albumin at 0.25 h, 3 days	n/a	Activation of astrocytes and microglia on 3 days	n/a	n/a
Adams et al., 2018	Mouse	CHI; RHI	Decreased CBF and CVR detected by MRI on 14 days	Reduced evoked neuronal responses without changes in neuronal density on 14 days	Activation of astrocytes on 14 days	n/a	n/a
Bharadwaj et al., 2018	Mouse	MFP	BBB disruption detected by HRP staining and nanoparticles at 3 h	n/a	n/a	n/a	n/a
Tagge et al., 2018	Mouse	Lateral CHI	BBB disruption detected by Evans blue, extravasated serum albumin, and DCE-MRI at 24 h	Neuronal damage on 1 day; Decreased neuronal density on 3–14 days	Activation of astrocytes and microglia on 1–3 days	Transient impairments in neurobehavioral response tests	n/a
Wendel et al., 2018	Mouse (juvenile)	CHI	Increased vessel density and length detected by IHC on 4–60 days	White matter disruption on 4–14 days	n/a	n/a	n/a
Yates et al., 2017	Rat	Weight drop; RHI	BBB disruption detected by Evan blue on 3 days	n/a	No activation of astrocytes and microglia on 4 days	Deficit in Foot-fault test on 1 day	Impairment in MWM on 4 days
Ichkova et al., 2020	Mouse (juvenile)	CHI	BBB disruption and altered cortical cerebrovascular reactivity detected by IgG staining on 1–3 days	Axonal pathology on 30 days; No neuronal loss on 1, 3, 7, 30 days	n/a	n/a	Deficit in open-field test on 1–30 days
Kane et al., 2012	Mouse	Weight drop; RHI	No BBB disruption by IgG staining on 7 days	n/a	Mild activation of astrocytes on 7 days	Deficit in rotarod test on 1 day and locomotor activity on 5 days	n/a
Meehan et al., 2012	Mouse	Weight drop; RHI	No BBB disruption by IgG staining at 1 h, 24 h	No changes in neuronal degeneration and axonal injury at 1 h, 24 h	n/a	n/a	Impairment in MWM at 1 month
Lynch et al., 2016	Mouse (aged)	CHI; RHI	No BBB disruption by S100β ELISA or occludin at 7 months	n/a	Activation of astrocytes and microglia in the CC	n/a	Spatial memory deficits in Barnes Maze task at 1–6 months
Chung et al., 2019	Mouse	CHI	No BBB disruption by IgG staining at 24 h	No changes in neuronal degeneration at 24 h, 48 h	Increased infiltration of CD11b^+^/CD45^+^ leukocytes at 72 h	No deficit in Foot-fault test on 1–14 days	Impairment in MWM on 3–42 days
Wu et al., 2019	Mouse (adolescent)	CHI; RHI	No BBB disruption by Evan blue at 4 h	No changes in neuronal degeneration at 1 year	No activation of astrocytes and microglia at 4 h–1 year	n/a	Impairment in MWM on 7 days–9 months

*BBB, blood–brain barrier; CBF, cerebral blood flow; CC, corpus callosum; CCI, controlled cortical impact; CHI, closed head injury; CVR, cerebrovascular reactivity; DCE-MRI, dynamic contrast-enhanced MRI; ECM, extracellular matrix; ELISA, enzyme-linked immunosorbent assay; FITC, fluorescein isothiocyanate; HRP, horseradish peroxidase; IHC, immunohistochemistry; LFP, lateral fluid percussion; MDC, macrophage-derived chemokine; MFP, midline fluid percussion; MIP-1α, macrophage inflammatory protein-1α; MRI, magnetic resonance imaging; mTBI, mild traumatic brain injury; MWM, Morris water maze; NBS, neurobehavioral scores; NSS, neurological severity scores; NOR, novel object recognition; NSE, neuron-specific enolase; RHI, repetitive head injury; SOR, spatial object recognition.*

We surveyed the BBB dysfunction and relevant pathologic changes found in mouse or rat models, covering the acute and subacute stages that evolve within the first 2 weeks after mTBI and the chronic stage that usually takes place 2 weeks after mTBI. The methods commonly used for BBB functional analysis in murine models are (i) histological assessment using plasma proteins such as immunoglobulin G (IgG) and/or exogenous tracers such as Evans blue dye, horseradish peroxidase (HRP), or fluoresce labeled albumin; (ii) *in vivo* imaging techniques including magnetic resonance imaging (MRI) and multi-photon imaging; and (iii) additional methods such as brain water content calculation for cerebral edema (wet/dry weight ratio). Analysis of the protein and mRNA expression of tight junction protein was also reported.

## Blood–Brain Barrier Dysfunction in Acute and Subacute Stages of Mild Traumatic Brain Injury

Twelve out of 17 of these mTBI studies showed that the BBB breakdown is an early event in murine models of mTBI, even as early as 5 min post-injury. For example, Li et al. (2016) examined the integrity of BBB in a modified CCI model of mTBI *via* the *in vivo* two-photon imaging of intravenously injected rhodamine B. They showed that BBB disruption in wild-type C57BL/6 mice occurred at a very early stage of mTBI (between 5 and 60 min), which was even exacerbated in Slit2-Tg mice (Li et al., 2016). Using peripherally injected radiotracer, ^14^C-sucrose and ^99m^Tc-albumin, Logsdon et al. (2018) found BBB disruption by mild blast exposure in just 15 min. Moreover, based on immunostaining of endogenous IgG, BBB disruption was detected in both adult and juvenile mice from 6 h to 2 days after mTBI (Laurer et al., 2001; Huh et al., 2008; Ichkova et al., 2020). By administering exogenous tracers Evans blue dye or fluoresce labeled albumin, the BBB breakdown was also detected from the mTBI between 1 and 24 h after mTBI based on extravascular leakages (Yang et al., 2013; Chen et al., 2014; Katz et al., 2015; Yates et al., 2017). Tagge et al. (2018) applied a novel closed-head concussive left-lateral impact injury mouse model to investigate the microvascular injury after mTBI. Both Evans blue extravasation and *in vivo* dynamic MRI of systemically administered gadolinium-based contrast agent confirmed acute and persistent BBB disruption in the ipsilateral cortex of impacted mice. They also examined the postmortem neuropathological changes and did not find the evidence of hemorrhagic contusion, suggesting BBB dysfunction rather than intraparenchymal hemorrhage resulted in permeability changes (Tagge et al., 2018). These data indicate that BBB breakdown occurs within minutes after mTBI; however, it may only be detected by more sensitive methods than classic histological analysis.

**FIGURE 3:**
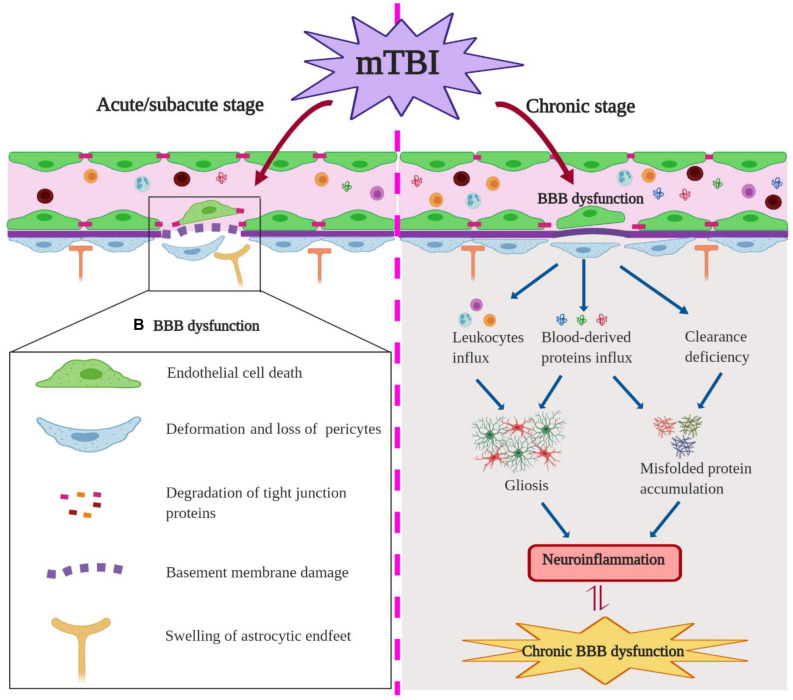
Blood–brain barrier (BBB) dysfunction in the acute/subacute stage **(Left)** and chronic stage **(Right)** of mild traumatic brain injury (mTBI). In the acute/subacute stage, mTBI induces the endothelial cell (EC) death, loss of pericytes and tight junction proteins, astrocytic endfeet swelling, as well as basement membrane damage and ultimately an impairment of BBB permeability. In the chronic stage, mTBI causes BBB leakage and influx of neurotoxic blood-derived proteins and cells into the brain parenchyma, which can induce gliosis and misfolded protein accumulation post-insult; mTBI-induced clearance deficiency of BBB promotes misfolded protein accumulation.

The mechanism of the BBB dysfunction after mTBI was also investigated in some of the studies. For example, reduced expression of tight junction protein claudin-5 and BBB disruption were detected in an mTBI model with a blast method, and inhibiting nitric oxide-dependent signaling pathways and preserving tight junction integrity were helpful to maintain BBB integrity after injury (Logsdon et al., 2018). mTBI-induced acute BBB disruption was also associated with microvascular structural damages including swollen astrocyte endfeet and deformation of pericytes in cortical regions 6 h post-injury (Bashir et al., 2020). Using a closed-head TBI model, Tagge et al. (2018) described that mTBI induced capillary retraction, changes in the extracellular matrix and basal lamina, and astrocytic endfeet engorgement, which all contribute to BBB disruption. Interestingly, these vascular structural changes such as loss of tight junctions and pericytes and swollen endfeet (Figure 3, left) are commonly seen in neurodegenerative diseases such as AD, suggesting that a shared underlying mechanism may exist in these distinct pathological conditions.

However, five recent studies found no evident BBB disruption after mTBI (Table 1). For example, Kane et al. (2012) found mild astrocytic activation but lack of BBB disruption to IgG or edema in weight drop model of mTBI in mice, while Whalen’s group reported that the BBB integrity was not compromised after single or repetitive closed head injury by immunohistochemical analysis of Evans blue or intracerebral levels of mouse IgG (Meehan et al., 2012; Chung et al., 2019; Wu et al., 2019). In a long-term study, Lynch et al. (2016) also found no BBB leakage to Evans blue at 7 months after repetitive midline mTBI, despite vascular abnormality still existed at that time. These different results may reflect the differences in animal models, methodology in detecting BBB changes, and observation time points.

## Blood–Brain Barrier Dysfunction in the Chronic Stage of Mild Traumatic Brain Injury

In addition to its breakdown during the acute and subacute phases of mTBI, the pathological changes of BBB integrity in the chronic phase of mTBI have been well documented in human patients, yet underexplored in animal models (Jullienne et al., 2016; Sandsmark et al., 2019). For example, Tomkins et al. (2011) found that 13 of 27 mTBI patients showed parenchymal regions with BBB disruption based on MRI scans, which were identified up to a median of 2.5 months after the initial trauma event, indicating that lasting BBB disruption exists in nearly half of the mTBI patients. In a different cohort of 17 mTBI patients with post-concussion syndromes (PCSs), eight of them exhibited abnormal BBB permeability based on single-photon emission computed tomography (SPECT) of ^99m^Tc-diethylenetriaminepentaacetic acid radiotracer (Korn et al., 2005). These patients were scanned between 1 month and 7 years after their respective head injuries. More recently, Yoo et al. (2019) demonstrated that mTBI patients with PCS, who had a median of 4-month time interval between injury and MRI examinations, exhibited much higher BBB permeability based on K^trans^ measurement from dynamic contrast-enhanced MRI (DCE-MRI) when compared with controls. Similar situations were also reported from patients diagnosed with concussion in adolescent sports (O’Keeffe et al., 2020) and football players (Weissberg et al., 2014).

Consistent with observations in human patients, cerebrovascular dysfunction was detected in a repeated mTBI mouse model at 2 weeks after the insult (Adams et al., 2018). Functional assessments revealed that repeated impacts cause sustained decreases of CBF and cerebrovascular reactivity, along with neuronal function deficits and astrogliosis in peri-contusion areas (Adams et al., 2018). In addition, mTBI can elicit an early and long-lasting cerebrovascular dysfunction in juvenile mice (Wendel et al., 2018; Ichkova et al., 2020), accompanied by astrocyte response and gliovascular changes (Rodriguez-Grande et al., 2018; Clément et al., 2020). Furthermore, pediatric mTBI can morphologically alter the vasculature of the ipsilateral corpus callosum differentially between the acute/subacute stage and the chronic stage (Wendel et al., 2018). As demonstrated, mTBI induced an initial increase in vessel parameters (e.g., vessel density and length) at 4 days post-injury (DPI), which was followed by a transient decrease at 14 DPI but with a subsequent increase in vessel density at 60 DPI (Wendel et al., 2018). It suggested that these microvascular alterations contribute to the long-term reorganization of the ipsilateral corpus callosum after mTBI (Wendel et al., 2018). Finally, chronic changes in the microvasculature were also reported in rats several months after the initial blast exposure (Gama Sosa et al., 2014). In particular, intraventricular hemorrhage was observed in four out of 23 blast-exposed animals examined between 6 and 10 months after the last blast exposure, which may be attributed to continued vascular fragility within the choroid plexus post-injury (Gama Sosa et al., 2014). As suggested, blast exposure may induce the degradation and remodeling of the extracellular matrix, which contributes to chronic microvascular pathology after injury (Gama Sosa et al., 2014). Due to the limited number of reports, the potential molecular and cellular mechanisms of BBB dysfunction in the chronic phase of murine mTBI remain largely unknown. Abnormal expressions of junctional proteins and matrix metallopeptidases may potentially involve the degradation of the extracellular matrix and prolonged BBB breakdown, and chronic inflammation may also play a crucial role. More importantly, the crosstalk between vascular impairment and neuroinflammation in the context of persistent BBB dysfunction after mTBI is yet to be determined (Figure 3, right).

## Blood–Brain Barrier Dysfunction of Mild Traumatic Brain Injury Model in the High-Throughput Sequencing Era

Over the past decade, high-throughput sequencing methods have revolutionized the entire field of biology. The RNA sequencing to study the entire transcriptomes in detail has driven many important discoveries for various neurological disorders (von Gertten et al., 2005; Redell et al., 2013; Lipponen et al., 2016; Meng et al., 2017). The brain microvascular endothelial cells are the major component of the BBB and play critical roles to maintain its normal function and integrity. Therefore, understanding endothelial cell-specific transcriptional profiles can help us to identify novel mechanisms of TBI-induced changes within the BBB. As most of the previous genomic profiling studies of TBI are based on heterogeneous mixtures of brain cell types, Munji et al. (2019) recently used endothelial cell enrichment for deep RNA sequencing to decipher the transcriptome differences at 24 h (acute), 72 h (subacute), and 1 month (chronic) after TBI in mice. They found that most unique and dramatic changes were at the acute time point, whereas few overlapped genes were observed between acute and chronic periods. These findings reflect the severity of the initial insult on the endothelial functions and BBB integrity and immediate response at the early stage, yet clearly point out to the distinct molecular mechanisms that are involved in acute/subacute and chronic phases of TBI. In addition, they also found that the synthesis of extracellular matrix molecules and activation of proteases further contributed to the BBB changes in the acute and subacute period. On the other hand, the immune response may play a more prominent role in the chronic period. These findings provided us further directions for investigating the endothelial dysfunction in TBI.

The diversity of cell types at the BBB (Vanlandewijck et al., 2018), only focusing on endothelial cells, will mask crucial signals from other BBB-related cell types. Therefore, single-cell RNA sequencing represents an approach to overcome this problem. Arneson et al. (2018) firstly investigated the mTBI pathogenesis in thousands of individual brain cells in parallel using single-cell RNA sequencing. Unsupervised clustering analysis identified BBB-associated cell types such as endothelial cells and mural cells. Besides, they also found a previously unknown cell type after mTBI, which likely is a migrating endothelial cell, as it carries key signatures of cell growth and migration as well as endothelial identity. This is consistent with previous research that endothelial cell is a main component of BBB that is destroyed after mTBI (Shlosberg et al., 2010), and the proliferation and migration of endothelial cells are inherent aspects of neovascularization after injuries (Salehi et al., 2017). More importantly, pathway analysis informed by genes with significant changes from both endothelial cells and astrocytes implicates endoplasmic reticulum dysfunction after mTBI. As endoplasmic reticulum stress is a key contributor to the injury-induced neurodegeneration (Oakes and Papa, 2015), the single-cell RNA sequencing data indicated that endoplasmic reticulum dysfunction in BBB may be an overlooked mechanism in mTBI. In addition, their data also demonstrated that cellular interactions based on extracellular matrix of endothelial cells are also heavily impacted after mTBI (Arneson et al., 2018), which is consistent with a recent report (Munji et al., 2019). Taken together, deep RNA sequencing and single-cell RNA sequencing data have become valuable resources to explore BBB dysfunction in mTBI, which will likely bring new insights to the true mechanism of vascular impairment after mTBI.

## Blood–Brain Barrier Dysfunction and Neuroinflammation in Mild Traumatic Brain Injury

In animal models of mTBI, BBB dysfunction is closely associated with neuroinflammation in both acute/subacute stage and chronic period. In the event of mTBI, the tight junction complexes and basement membrane are disrupted, which results in increased permeability of BBB and inflammatory response after injury (Chodobski et al., 2011). Activation of microglia, stimulation of astrocytes, and neuronal cell death are closely associated with neurodegeneration after mTBI (Ramos-Cejudo et al., 2018; Figure 1). Notably, many studies reported that microglia and astrocytes are activated in the acute period after mTBI. For example, in a lateral impact of closed head injury mouse model, Tagge et al. (2018) reported that activated microglia and reactive astrocytes were detected at 24 h post-injury in the ipsilateral cortex, which peaked around 3 DPI and started to be resolved in 2 weeks, although perivascular accumulation of hemosiderin-laden macrophages may persist.

Limited evidence has indicated that BBB dysfunction precedes gliosis and neuroinflammation at least in murine models of mTBI. For example, Huh et al. (2008) showed that BBB disruption in their midline CCI rat model of mTBI occurs within 6 h after impact, while glial fibrillary acidic protein (GFAP) immunoreactivity in the cortex at 24 h was comparable to that observed in sham-injured animals, and astrogliosis was only observed on day 3 and day 8 post-injury. As BBB breakdown and extravasation of plasma proteins such as fibrinogen are a driving force of microglia activation after injury (Davalos et al., 2012) and capable of inducing neurotoxic reactive astrocytes after TBI (Liddelow and Barres, 2017; Liddelow et al., 2017) and cognitive impairment (Fulop et al., 2019), the vascular link between mTBI and neuroinflammation should be defined in future studies.

## Blood–Brain Barrier Dysfunction and Cognitive Impairment in Murine Mild Traumatic Brain Injury Models

Among the 17 articles of BBB dysfunction in mTBI, nine of them studied whether mTBI-induced BBB dysfunction was related to cognitive impairment in mice and rats. Meehan et al. (2012) and Yates et al. (2017) used a weight drop model of mTBI as well as Huh et al. (2008) used a midline brain injury model and found that mTBI induced BBB dysfunction and significant reduction in Morris water maze (MWM) performance from injured mice and rats when compared with sham-operated controls. Yang et al. (2013) also found that mTBI mice with BBB impairment spent significantly less time in investigating a novel object during the novel object recognition test in the first few days. In addition, Katz et al. (2015) used a lateral fluid percussion model to induce mTBI in rats and noted that mTBI rats displayed significant BBB leakage as well as acute cognitive impairment. These injured rats exhibited significantly lower neurological severity score (NSS) and neurobehavioral score (NBS) at 2 and 24 h after injury (Katz et al., 2015). Additionally, Ichkova et al. (2020) found that an early cerebrovascular pathology including BBB disruption may contribute to long-term behavioral deficits in mice following experimental juvenile mTBI, while the exact topographical coherence and the direct causality between these two events require further investigation.

However, Chen et al. (2014) and Laurer et al. (2001) found mTBI caused no significant impairments in either acute or long-term cognitive ability in the CCI models, although BBB dysfunction was reported in these animals. On the other hand, although Kane et al. (2012) and Meehan et al. (2012) found motor and cognitive deficits in mTBI animals, no BBB disruption was observed. While the different injury methodologies between groups may underlie the discrepancies in behavioral outcomes, further studies should still address the link between BBB dysfunction and cognitive impairment in animal models of mTBI.

## Discussion

Mild traumatic brain injury commonly occurs in professional sports (such as American football and boxing) and military service, which can be exacerbated by repetitive mild trauma injury (Baker and Patel, 2000). There are growing interests to investigate the acute/subacute and long-term pathologic changes after mTBI, as well as a focus on motor and cognitive impairments. In this study, we collected preclinical evidence from murine models to describe the role of BBB dysfunction in mTBI. BBB plays a key role in maintaining brain function stability, the integrity of BBB may be compromised under pathologic conditions such as TBI, stroke, brain tumor, and AD (Sweeney et al., 2019). For example, previous research showed BBB leakage was detected in a stroke model of rat (Michalski et al., 2010). In our review, we included 17 representative studies that described the BBB breakdown after mTBI. Twelve of them reported that the BBB was compromised after mTBI; however, five studies indicated that BBB breakdown was not detected in mTBI, even in receptive mTBI models. Up to now, it is still unclear whether the minor discrepancy was a result of the differences in mTBI animal models, animal ages, procedures, time points of observation, or methodology.

Mechanistically, impairment of BBB can initiate a series of adverse events, including the leakage of serum-derived circulating agents into the brain parenchyma and improper activation of signaling pathways (Manev, 2009). As some studies indicated that mTBI can induce sustained shear stress located within the impact zone, capillary retraction, pericyte degeneration, and astrocytic endfeet swelling, which all contribute to microvascular injury and BBB breakdown post-insult (Tagge et al., 2018), the exact mechanism of BBB impairment in different models of mTBI could still vary and depend on the severity. Importantly, emerging evidence from human genome-wide association studies suggests that many signature genes and network regulators of TBI may be associated with neurological disorders, which could be used as elements of prognosis and plausible interventional targets for TBI (Meng et al., 2017). Single-cell molecular alterations were reported after mTBI by using unbiased single-cell sequencing, which provides new insights to the molecular pathway mechanism and therapeutics in mTBI and its related neurodegeneration (Arneson et al., 2018).

Mild traumatic brain injury is considered a long-term risk for neurobehavioral changes, cognitive decline, and neurodegenerative disease including AD (McAllister and McCrea, 2017). Cognitive and motor function changes commonly occur after mTBI and may have lifelong consequences, which are still difficult to detect and trace in clinical settings. On the other hand, murine animal models of mTBI provide us quantitative measures and longitudinal follow-ups. Most used methods for motor function tests include Foot-fault, rotarod, and beam walking assays, and cognitive function tests include nest construction, food burrowing, novel object recognition, fear conditioning, MWM, etc. In our review, we found that most studies reported the motor deficit and cognitive impairment after mTBI range from 1 day to several months. However, few studies indicated the absence of motor or cognitive function impairment, which may be due to differences in animal models or procedures as described above. Vascular dysfunction and BBB breakdown are associated with cognitive impairments in aging and neurodegenerative diseases such as AD (Iadecola, 2004). In the mTBI event, cerebrovascular dysfunction can result in circulatory insufficiency and cause neuronal dysfunction (Jullienne et al., 2016). Edema formation and BBB breakdown after mTBI can also disturb the brain homeostasis and the clearance of toxic products such as β-amyloid, which will accelerate the neuronal damage and contribute to the mTBI-associated late-life neurodegeneration. Can we target vascular dysfunctions in mTBI as a potential therapeutic intervention? Histological and genetic profiling evidence has indicated changes in different BBB modalities after mTBI (Meng et al., 2017; Arneson et al., 2018; Munji et al., 2019; Sandsmark et al., 2019), including alteration of extracellular matrix and basement membrane, metalloproteinase, etc. Targeting these endophenotypes may offer novel therapeutic opportunities. But the journey is still out there.

## Conclusion

In this review, we focused on BBB dysfunction after mTBI in murine models and found that BBB breakdown and microvascular impairment are important pathological mechanisms for cognitive impairment after mTBI. Restoring vascular functions might be beneficial for patients with mTBI and reduce the risk of developing cognitive impairments post-insult.

## Author Contributions

ZZ and YW designed the review outline, wrote and reviewed the review, did the literature search, and data extraction and interpretation. HW, XG, and BP provided critical reviews, revised the manuscript, and provided relevant insights and edits. All authors read and approved the final version of the manuscript.

## Conflict of Interest

The authors declare that the research was conducted in the absence of any commercial or financial relationships that could be construed as a potential conflict of interest.

## References

[B1] Adams C. Bazzigaluppi P. Beckett T. L. Bishay J. Weisspapir I. Dorr A. (2018). Neurogliovascular dysfunction in a model of repeated traumatic brain injury. *Theranostics* 8 4824–4836. 10.7150/thno.24747 30279740 PMC6160760

[B2] Arneson D. Zhang G. Ying Z. Zhuang Y. Byun H. R. Ahn I. S. (2018). Single cell molecular alterations reveal target cells and pathways of concussive brain injury. *Nat. Commun.* 9:3894. 10.1038/s41467-018-06222-0 30254269 PMC6156584

[B3] Baker R. J. Patel D. R. (2000). Sports related mild traumatic brain injury in adolescents. *Indian J. Pediatr.* 67 317–321. 10.1007/bf02820676 10885200

[B4] Bashir A. Abebe Z. A. McInnes K. A. Button E. B. Tatarnikov I. Cheng W. H. (2020). Increased severity of the CHIMERA model induces acute vascular injury, sub-acute deficits in memory recall, and chronic white matter gliosis. *Exp. Neurol.* 324:113116. 10.1016/j.expneurol.2019.113116 31734317

[B5] Bharadwaj V. N. Rowe R. K. Harrison J. Wu C. Anderson T. R. Lifshitz J. (2018). Blood–brain barrier disruption dictates nanoparticle accumulation following experimental brain injury. *Nanomedicine* 14, 2155–2166. 10.1016/j.nano.2018.06.004 29933022 PMC6177306

[B6] Bhowmick S. D’Mello V. Caruso D. Wallerstein A. Abdul-Muneer P. M. (2019). Impairment of pericyte-endothelium crosstalk leads to blood-brain barrier dysfunction following traumatic brain injury. *Exp. Neurol.* 317 260–270. 10.1016/j.expneurol.2019.03.014 30926390

[B7] Blennow K. Hardy J. Zetterberg H. (2012). The neuropathology and neurobiology of traumatic brain injury. *Neuron* 76 886–899. 10.1016/j.neuron.2012.11.021 23217738

[B8] Bodnar C. N. Roberts K. N. Higgins E. K. Bachstetter A. D. (2019). A systematic review of closed head injury models of mild traumatic brain injury in mice and rats. *J. Neurotrauma* 36 1683–1706. 10.1089/neu.2018.6127 30661454 PMC6555186

[B9] Chamelian L. Feinstein A. (2004). Outcome after mild to moderate traumatic brain injury: the role of dizziness. *Arch. Phys. Med. Rehabil.* 85 1662–1666. 10.1016/j.apmr.2004.02.012 15468028

[B10] Chen Y. Mao H. Yang K. H. Abel T. Meaney D. F. (2014). A modified controlled cortical impact technique to model mild traumatic brain injury mechanics in mice. *Front. Neurol.* 5:100. 10.3389/fneur.2014.00100 24994996 PMC4061598

[B11] Chodobski A. Zink B. J. Szmydynger-Chodobska J. (2011). Blood-brain barrier pathophysiology in traumatic brain injury. *Transl. Stroke Res.* 2 492–516. 10.1007/s12975-011-0125-x 22299022 PMC3268209

[B12] Chung J. Y. Krapp N. Wu L. Lule S. McAllister L. M. Edmiston W. J. (2019). Interleukin-1 receptor 1 deletion in focal and diffuse experimental traumatic brain injury in mice. *J. Neurotrauma* 36 370–379. 10.1089/neu.2018.5659 29768967 PMC6338578

[B13] Clément T. Lee J. B. Ichkova A. Rodriguez-Grande B. Fournier M.-L. Aussudre J. (2020). Juvenile mild traumatic brain injury elicits distinct spatiotemporal astrocyte responses. *Glia* 68 528–542. 10.1002/glia.23736 31670865

[B14] Daneman R. Prat A. (2015). The blood-brain barrier. *Cold Spring Harb. Perspect. Biol.* 7:a020412. 10.1101/cshperspect.a020412 25561720 PMC4292164

[B15] Davalos D. Kyu Ryu J. Merlini M. Baeten K. M. Le Moan N. Petersen M. A. (2012). Fibrinogen-induced perivascular microglial clustering is required for the development of axonal damage in neuroinflammation. *Nat. Commun.* 3:1227. 10.1038/ncomms2230 23187627 PMC3514498

[B16] Doherty C. P. O’Keefe E. Wallace E. Loftus T. Keaney J. Kealy J. (2016). Blood-brain barrier dysfunction as a hallmark pathology in chronic traumatic encephalopathy. *J. Neuropathol. Exp. Neurol.* 75 656–662. 10.1093/jnen/nlw036 27245243 PMC4913433

[B17] Fulop G. A. Ahire C. Csipo T. Tarantini S. Kiss T. Balasubramanian P. (2019). Cerebral venous congestion promotes blood-brain barrier disruption and neuroinflammation, impairing cognitive function in mice. *Geroscience* 41 575–589. 10.1007/s11357-019-00110-1 31691147 PMC6885079

[B18] Gama Sosa M. A. De Gasperi R. Janssen P. L. Yuk F. J. Anazodo P. C. Pricop P. E. (2014). Selective vulnerability of the cerebral vasculature to blast injury in a rat model of mild traumatic brain injury. *Acta Neuropathol. Commun.* 2:67. 10.1186/2051-5960-2-67 24938728 PMC4229875

[B19] Hackenberg K. Unterberg A. (2016). [Traumatic brain injury]. *Nervenarzt* 87 203–216. 10.1007/s00115-015-0051-3 26810405

[B20] Han X. Chai Z. Ping X. Song L.-J. Ma C. Ruan Y. (2020). In vivo two-photon imaging reveals acute cerebral vascular spasm and microthrombosis after mild traumatic brain injury in mice. *Front. Neurosci.* 14:210. 10.3389/fnins.2020.00210 32210758 PMC7077429

[B21] Huh J. W. Widing A. G. Raghupathi R. (2008). Midline brain injury in the immature rat induces sustained cognitive deficits, bihemispheric axonal injury and neurodegeneration. *Exp. Neurol.* 213 84–92. 10.1016/j.expneurol.2008.05.009 18599043 PMC2633731

[B22] Iadecola C. (2004). Neurovascular regulation in the normal brain and in Alzheimer’s disease. *Nat. Rev. Neurosci.* 5 347–360. 10.1038/nrn1387 15100718

[B23] Ichkova A. Rodriguez-Grande B. Zub E. Saudi A. Fournier M.-L. Aussudre J. (2020). Early cerebrovascular and long-term neurological modifications ensue following juvenile mild traumatic brain injury in male mice. *Neurobiol. Dis.* 141:104952. 10.1016/j.nbd.2020.104952 32442681

[B24] Jullienne A. Obenaus A. Ichkova A. Savona-Baron C. Pearce W. J. Badaut J. (2016). Chronic cerebrovascular dysfunction after traumatic brain injury. *J. Neurosci. Res.* 94 609–622. 10.1002/jnr.23732 27117494 PMC5415378

[B25] Kane M. J. Angoa-Pérez M. Briggs D. I. Viano D. C. Kreipke C. W. Kuhn D. M. (2012). A mouse model of human repetitive mild traumatic brain injury. *J. Neurosci. Methods* 203 41–49. 10.1016/j.jneumeth.2011.09.003 21930157 PMC3221913

[B26] Katz P. S. Sulzer J. K. Impastato R. A. Teng S. X. Rogers E. K. Molina P. E. (2015). Endocannabinoid degradation inhibition improves neurobehavioral function, blood-brain barrier integrity, and neuroinflammation following mild traumatic brain injury. *J. Neurotrauma* 32 297–306. 10.1089/neu.2014.3508 25166905 PMC4348366

[B27] Korn A. Golan H. Melamed I. Pascual-Marqui R. Friedman A. (2005). Focal cortical dysfunction and blood-brain barrier disruption in patients with Postconcussion syndrome. *J. Clin. Neurophysiol.* 22 1–9. 10.1097/01.wnp.0000150973.24324.a7 15689708

[B28] Laurer H. L. Bareyre F. M. Lee V. M. Trojanowski J. Q. Longhi L. Hoover R. (2001). Mild head injury increasing the brain’s vulnerability to a second concussive impact. *J. Neurosurg.* 95 859–870. 10.3171/jns.2001.95.5.0859 11702878

[B29] Li S. Li H. He X.-F. Li G. Zhang Q. Liang F.-Y. (2016). Transgenic over-expression of slit2 enhances disruption of blood-brain barrier and increases cell death after traumatic brain injury in mice. *Neurosci. Lett.* 631 85–90. 10.1016/j.neulet.2016.08.013 27521753

[B30] Liddelow S. A. Barres B. A. (2017). Reactive astrocytes: production, function, and therapeutic potential. *Immunity* 46 957–967. 10.1016/j.immuni.2017.06.006 28636962

[B31] Liddelow S. A. Guttenplan K. A. Clarke L. E. Bennett F. C. Bohlen C. J. Schirmer L. (2017). Neurotoxic reactive astrocytes are induced by activated microglia. *Nature* 541 481–487. 10.1038/nature21029 28099414 PMC5404890

[B32] Lifshitz J. Rowe R. K. Griffiths D. R. Evilsizor M. N. Thomas T. C. Adelson P. D. (2016). Clinical relevance of midline fluid percussion brain injury: acute deficits, chronic morbidities and the utility of biomarkers. *Brain Injury* 30 1293–1301. 10.1080/02699052.2016.1193628 27712117 PMC5303557

[B33] Lipponen A. Paananen J. Puhakka N. Pitkänen A. (2016). Analysis of post-traumatic brain injury gene expression signature reveals tubulins, Nfe2l2, Nfkb, Cd44, and S100a4 as treatment targets. *Sci. Rep.* 6:31570. 10.1038/srep31570 27530814 PMC4987651

[B34] Logsdon A. F. Meabon J. S. Cline M. M. Bullock K. M. Raskind M. A. Peskind E. R. (2018). Blast exposure elicits blood-brain barrier disruption and repair mediated by tight junction integrity and nitric oxide dependent processes. *Sci. Rep.* 8:11344. 10.1038/s41598-018-29341-6 30054495 PMC6063850

[B35] Lynch C. E. Crynen G. Ferguson S. Mouzon B. Paris D. Ojo J. (2016). Chronic cerebrovascular abnormalities in a mouse model of repetitive mild traumatic brain injury. *Brain Injury* 30 1414–1427. 10.1080/02699052.2016.1219060 27834539

[B36] Manev H. (2009). Hypotheses on mechanisms linking cardiovascular and psychiatric/neurological disorders. *Cardiovasc. Psychiatry Neurol.* 2009:197132. 10.1155/2009/197132 20029617 PMC2790123

[B37] McAllister T. McCrea M. (2017). Long-term cognitive and neuropsychiatric consequences of repetitive concussion and head-impact exposure. *J. Athl. Train* 52 309–317. 10.4085/1062-6050-52.1.14 28387556 PMC5384827

[B38] Meehan W. P. Zhang J. Mannix R. Whalen M. J. (2012). Increasing recovery time between injuries improves cognitive outcome after repetitive mild concussive brain injuries in mice. *Neurosurgery* 71 885–891. 10.1227/NEU.0b013e318265a439 22743360 PMC5815628

[B39] Meng Q. Zhuang Y. Ying Z. Agrawal R. Yang X. Gomez-Pinilla F. (2017). Traumatic brain injury induces genome-wide transcriptomic, methylomic, and network perturbations in brain and blood predicting neurological disorders. *EBioMedicine* 16 184–194. 10.1016/j.ebiom.2017.01.046 28174132 PMC5474519

[B40] Michalski D. Grosche J. Pelz J. Schneider D. Weise C. Bauer U. (2010). A novel quantification of blood-brain barrier damage and histochemical typing after embolic stroke in rats. *Brain Res.* 1359 186–200. 10.1016/j.brainres.2010.08.045 20732314

[B41] Munji R. N. Soung A. L. Weiner G. A. Sohet F. Semple B. D. Trivedi A. (2019). Profiling the mouse brain endothelial transcriptome in health and disease models reveals a core blood–brain barrier dysfunction module. *Nat. Neurosci.* 22 1892–1902. 10.1038/s41593-019-0497-x 31611708 PMC6858546

[B42] Oakes S. A. Papa F. R. (2015). The role of endoplasmic reticulum stress in human pathology. *Annu. Rev. Pathol. Mech. Dis.* 10 173–194. 10.1146/annurev-pathol-012513-104649 25387057 PMC5568783

[B43] O’Keeffe E. Kelly E. Liu Y. Giordano C. Wallace E. Hynes M. (2020). Dynamic blood-brain barrier regulation in mild traumatic brain injury. *J. Neurotrauma* 37 347–356. 10.1089/neu.2019.6483 31702476 PMC10331162

[B44] Ramos-Cejudo J. Wisniewski T. Marmar C. Zetterberg H. Blennow K. de Leon M. J. (2018). Traumatic brain injury and Alzheimer’s disease: the cerebrovascular link. *EBioMedicine* 28 21–30. 29396300 10.1016/j.ebiom.2018.01.021 PMC5835563

[B45] Redell J. B. Moore A. N. Grill R. J. Johnson D. Zhao J. Liu Y. (2013). Analysis of functional pathways altered after mild traumatic brain injury. *J. Neurotrauma* 30 752–764. 10.1089/neu.2012.2437 22913729 PMC3653386

[B46] Riggio S. (2011). Traumatic brain injury and its neurobehavioral sequelae. *Neurol. Clin.* 29 35–47. 10.1016/j.ncl.2010.10.008 21172569

[B47] Rodriguez-Grande B. Obenaus A. Ichkova A. Aussudre J. Bessy T. Barse E. (2018). Gliovascular changes precede white matter damage and long-term disorders in juvenile mild closed head injury. *Glia* 66 1663–1677. 10.1002/glia.23336 29665077

[B48] Rutland-Brown W. Langlois J. A. Thomas K. E. Xi Y. L. (2006). Incidence of traumatic brain injury in the United States, 2003. *J. Head Trauma Rehabil.* 21 544–548. 10.1097/00001199-200611000-00009 17122685

[B49] Salehi A. Zhang J. H. Obenaus A. (2017). Response of the cerebral vasculature following traumatic brain injury. *J. Cereb. Blood Flow Metab.* 37 2320–2339. 10.1177/0271678X17701460 28378621 PMC5531360

[B50] Sandsmark D. K. Bashir A. Wellington C. L. Diaz-Arrastia R. (2019). Cerebral microvascular injury: a potentially treatable endophenotype of traumatic brain injury-induced neurodegeneration. *Neuron* 103 367–379. 10.1016/j.neuron.2019.06.002 31394062 PMC6688649

[B51] Shlosberg D. Benifla M. Kaufer D. Friedman A. (2010). Blood-brain barrier breakdown as a therapeutic target in traumatic brain injury. *Nat. Rev. Neurol.* 6 393–403. 10.1038/nrneurol.2010.74 20551947 PMC3625732

[B52] Sweeney M. D. Zhao Z. Montagne A. Nelson A. R. Zlokovic B. V. (2019). Blood-brain barrier: from physiology to disease and back. *Physiol. Rev.* 99 21–78. 10.1152/physrev.00050.2017 30280653 PMC6335099

[B53] Tagge C. A. Fisher A. M. Minaeva O. V. Gaudreau-Balderrama A. Moncaster J. A. Zhang X.-L. (2018). Concussion, microvascular injury, and early tauopathy in young athletes after impact head injury and an impact concussion mouse model. *Brain* 141 422–458. 10.1093/brain/awx350 29360998 PMC5837414

[B54] Tomkins O. Feintuch A. Benifla M. Cohen A. Friedman A. Shelef I. (2011). Blood-brain barrier breakdown following traumatic brain injury: a possible role in posttraumatic epilepsy. *Cardiovasc. Psychiatry Neurol.* 2011:765923. 10.1155/2011/765923 21436875 PMC3056210

[B55] Vanlandewijck M. He L. Mäe M. A. Andrae J. Ando K. Del Gaudio F. (2018). A molecular atlas of cell types and zonation in the brain vasculature. *Nature* 554 475–480. 10.1038/nature25739 29443965

[B56] Villalba N. Sackheim A. M. Nunez I. A. Hill-Eubanks D. C. Nelson M. T. Wellman G. C. (2017). Traumatic Brain Injury Causes Endothelial Dysfunction in the Systemic Microcirculation through Arginase-1-Dependent Uncoupling of Endothelial Nitric Oxide Synthase. *J. Neurotrauma* 34 192–203. 10.1089/neu.2015.4340 26757855 PMC5198065

[B57] von Gertten C. Flores Morales A. Holmin S. Mathiesen T. Nordqvist A.-C. S. (2005). Genomic responses in rat cerebral cortex after traumatic brain injury. *BMC Neurosci.* 6:69. 10.1186/1471-2202-6-69 16318630 PMC1310614

[B58] Weissberg I. Veksler R. Kamintsky L. Saar-Ashkenazy R. Milikovsky D. Z. Shelef I. (2014). Imaging blood-brain barrier dysfunction in football players. *JAMA Neurol.* 71 1453–1455. 10.1001/jamaneurol.2014.2682 25383774

[B59] Wendel K. M. Lee J. B. Affeldt B. M. Hamer M. Harahap-Carrillo I. S. Pardo A. C. (2018). Corpus callosum vasculature predicts white matter microstructure abnormalities after pediatric mild traumatic brain injury. *J. Neurotrauma* [Online ahead of print]. 10.1089/neu.2018.5670 29739276

[B60] Wu L. Chung J. Y. Saith S. Tozzi L. Buckley E. M. Sanders B. (2019). Repetitive head injury in adolescent mice: a role for vascular inflammation. *J. Cereb. Blood Flow Metab.* 39 2196–2209. 10.1177/0271678X18786633 30001646 PMC6827111

[B61] Yang S. H. Gustafson J. Gangidine M. Stepien D. Schuster R. Pritts T. A. (2013). A murine model of mild traumatic brain injury exhibiting cognitive and motor deficits. *J. Surg. Res.* 184 981–988. 10.1016/j.jss.2013.03.075 23622728 PMC4073786

[B62] Yates N. J. Lydiard S. Fehily B. Weir G. Chin A. Bartlett C. A. (2017). Repeated mild traumatic brain injury in female rats increases lipid peroxidation in neurons. *Exp. Brain Res.* 235 2133–2149. 10.1007/s00221-017-4958-8 28417146

[B63] Yoo R.-E. Choi S. H. Oh B.-M. Do Shin S. Lee E. J. Shin D. J. (2019). Quantitative dynamic contrast-enhanced MR imaging shows widespread blood-brain barrier disruption in mild traumatic brain injury patients with post-concussion syndrome. *Eur. Radiol.* 29 1308–1317. 10.1007/s00330-018-5656-z 30066251

[B64] Zehendner C. M. Sebastiani A. Hugonnet A. Bischoff F. Luhmann H. J. Thal S. C. (2015). Traumatic brain injury results in rapid pericyte loss followed by reactive pericytosis in the cerebral cortex. *Sci. Rep.* 5:13497. 10.1038/srep13497 26333872 PMC4558600

[B65] Zhao Z. Nelson A. R. Betsholtz C. Zlokovic B. V. (2015). Establishment and dysfunction of the blood-brain barrier. *Cell* 163 1064–1078. 10.1016/j.cell.2015.10.067 26590417 PMC4655822

